# Simple and Fast DNA Based Sensor System for Screening of Small-Molecule Compounds Targeting Eukaryotic Topoisomerase 1

**DOI:** 10.3390/pharmaceutics13081255

**Published:** 2021-08-13

**Authors:** Kamilla Vandsø Petersen, Asier Selas, Kirstine Mejlstrup Hymøller, Karol Mizielinski, Maria Thorsager, Magnus Stougaard, Concepcion Alonso, Francisco Palacios, Yolanda Pérez-Pertejo, Rosa M. Reguera, Rafael Balaña-Fouce, Birgitta R. Knudsen, Cinzia Tesauro

**Affiliations:** 1 Department of Molecular Biology and Genetics, Aarhus University, 8000 Aarhus, Denmark; kvp@clin.au.dk (K.V.P.); khy@mbg.au.dk (K.M.H.); brk@mbg.au.dk (B.R.K.); 2 Department of Clinical Medicine, Aarhus University, 8000 Aarhus, Denmark; magnus.stougaard@clin.au.dk; 3 Department of Organic Chemistry, University of Basque Country (UPV/EHU), 01006 Vitoria-Gasteiz, Spain; asier.selas@ehu.eus (A.S.); concepcion.alonso@ehu.eus (C.A.); francisco.palacios@ehu.eus (F.P.); 4 VPCIR Biosciences ApS., 8000 Aarhus, Denmark; km@vpcir.com (K.M.); mt@vpcir.com (M.T.); 5 Department of Pathology, Aarhus University Hospital, 8000 Aarhus, Denmark; 6 Department of Biomedical Sciences, University of Leon (ULE), 24071 Leon, Spain; myperp@unileon.es (Y.P.-P.); rmregt@unileon.es (R.M.R.); rbalf@unileon.es (R.B.-F.)

**Keywords:** topoisomerase 1, REEAD, rolling circle amplification, small-molecule compounds, drug screening, enzyme activity, colorimetric readout

## Abstract

**Background**: Eukaryotic topoisomerase 1 is a potential target of anti-parasitic and anti-cancer drugs. Parasites require topoisomerase 1 activity for survival and, consequently, compounds that inhibit topoisomerase 1 activity may be of interest. All effective topoisomerase 1 drugs with anti-cancer activity act by inhibiting the ligation reaction of the enzyme. Screening for topoisomerase 1 targeting drugs, therefore, should involve the possibility of dissecting which step of topoisomerase 1 activity is affected. **Methods**: Here we present a novel DNA-based assay that allows for screening of the effect of small-molecule compounds targeting the binding/cleavage or the ligation steps of topoisomerase 1 catalysis. This novel assay is based on the detection of a rolling circle amplification product generated from a DNA circle resulting from topoisomerase 1 activity. **Results**: We show that the binding/cleavage and ligation reactions of topoisomerase 1 can be investigated separately in the presented assay termed REEAD (C|L) and demonstrate that the assay can be used to investigate, which of the individual steps of topoisomerase 1 catalysis are affected by small-molecule compounds. The assay is gel-free and the results can be detected by a simple colorimetric readout method using silver-on-gold precipitation rendering large equipment unnecessary. **Conclusion**: REEAD (C|L) allows for easy and quantitative investigations of topoisomerase 1 targeting compounds and can be performed in non-specialized laboratories.

## 1. Introduction

Rapid, reliable, and highly sensitive assays are essential for the development of novel compounds with potential clinical relevance. Both eukaryotic and prokaryotic DNA topoisomerases are among the DNA binding enzymes that attract high interest, because they are targets of small-molecule drugs with potential activity against human cancers or pathogens e.g., eukaryotic parasites or bacteria causing diseases in humans [1,2,3,4,5]. The biological function of DNA topoisomerases is to remove superhelical tension in genomic DNA. Thereby, they play essential roles in DNA replication, transcription, chromosome segregation, and recombination [6,7].

In eukaryotic cells, the type IB topoisomerase, topoisomerase 1 (TOP1) is an interesting target of anti-cancer [8,9,10] or anti-parasitic drugs [1,2]. This enzyme removes DNA topological tension by introducing transient single-strand nicks in double-stranded DNA in a reaction that involves the formation of a cleavage intermediate in which the enzyme is covalently attached to the 3′-phosphate end generated during cleavage. This allows for the free 5′-OH DNA end to rotate around the uncut strand to resolve topological stress in the DNA. After relaxation, the transient DNA nick is resealed in the religation reaction and the enzyme leaves the DNA intact [7,11].

TOP1 can be targeted by small-molecule compounds that act in very different manners. One class termed TOP1 inhibitors prevents the catalytic activity by inhibiting the DNA binding and/or cleavage of the enzyme. For several single cell eukaryotes such as the human pathogens *Trypanosoma brucei* and *Leishmania major* a largely reduced TOP1 activity level is detrimental [12,13] while in higher eukaryotes and in mammals, TOP1 appears only to be essential during the early developmental stages [14,15]. Hence, TOP1 inhibitors are of particular interest as potential anti-parasitic drugs [2]. The other class of TOP1 targeting compounds is termed poisons and acts specifically by prolonging the half-life of the cleavage intermediate. This can be accomplished by inhibiting the religation step of catalysis. Compounds of this type cause accumulation of TOP1 bound nicks in the genome leading to permanent DNA damage and cell death upon collision with DNA tracking processes such as transcription and replication. Consequently, this type of compound is of particular interest as anti-cancer drugs and indeed all known TOP1 targeting anti-cancer drugs including the water-soluble CPT derivatives, topotecan (TPT) and Irinotecan, belong to this family of drugs [9].

To identify TOP1 targeting poisons it is important to have access to easy-to-perform and fast assays that allow the mechanism-of-action of new compounds to be dissected. To this end, a number of in vitro assays, including relaxation assays [16], electrophoretic mobility shift assays (EMSA) [17] and DNA suicide cleavage–ligation assays [18,19,20,21] have been developed over the years. These assays can be used to access the inhibitory effect of new compounds as well as to elucidate which step(s) of TOP1 catalysis is affected. However, they all rely on gel-electrophoresis and require special equipment and expertise. Moreover, most of these assays only perform optimally when using relatively large amounts of at least partly purified TOP1 enzyme.

To enable assaying TOP1 activity in small and even crude samples, we previously developed a new type of TOP1 activity assay, the so-called Rolling circle Enhanced Enzyme Activity Detection (REEAD) assay [22]. In this assay the TOP1 cleavage–ligation activities convert a partly double-stranded DNA substrate to a closed DNA circle. This reaction can be performed in solution and the generated circle can subsequently be hybridized to a surface-attached primer to support Rolling Circle Amplification (RCA) leading to ~10^3^ tandem repeat products that can be visualized at the single molecule level in a fluorescence microscope upon hybridization to fluorescent labeled probes [22,23]. Alternatively, the substrates can be hybridized to the surface-attached primers before reaction with TOP1 in the so-called REEAD-on-a-slide setup [24]. In either case, since each cleavage–ligation reaction generates one DNA circle that results in one detectable product, the assay is highly sensitive, and it is directly quantitative. Moreover, since the conversion of the substrate to a closed circle relies specifically on the activity of TOP1, the assay can be used to measure enzyme activity and assess drug efficiency even in small crude biological samples [25,26,27,28]. Using this basic assay setup, we successfully measured TOP1 activity in single human cells [24] and predicted the CPT cytotoxicity in cancer cell lines [26]. Additionally, we measured TOP1 activity in biopsies from cancer patients [29] and proved it possible specifically to detect the presence of human pathogens in crude clinical samples [30,31].

In principle, the quantitative detection of the activity of TOP1 from humans or from human pathogens enabled by REEAD provides an attractive platform for screening of new compounds with potential clinical relevance for treatment of e.g., cancer or infectious diseases. However, while the original REEAD setup allows for measuring the effect of small-molecule compounds on the combined cleavage–ligation reaction of TOP1 it does not allow to determine which of the catalytic steps are affected and, hence, to classify potential new drugs into the inhibitor or the poison category. This is particularly important in the search of new anti-cancer drugs, where only poisons have proven effective so far [8,9]. To allow for screening of new compounds with potential inhibitor or poison effect, we here present a new REEAD-on-a-slide based setup (termed REEAD (C|L) in the following) that allows the effect of new compounds to be investigated on the separate binding/cleavage and religation steps of TOP1 catalysis. Note that non-covalent binding and cleavage is investigated simultaneously since these steps are not easy to separate using wild type TOP1 [32]. We demonstrate the feasibility of the system with the well characterized drug CPT as well as a less characterized new small-molecule compound, a (4-Phenyl-2-(4-trifluoromethylphenyl)-1,2,3,4-tetrahydroquinolin-8-yl) diphenylphosphine oxide, termed POTHQ [33]. Additionally, as an easy alternative to the more troublesome microscopic readout of REEAD signals described previously, we present a novel colorimetric detection system for the REEAD setup that is suitable for screening of small-molecule compounds and easily accessible for all laboratories.

## 2. Materials and Methods

### 2.1. Reagents

All chemicals were purchased from Sigma Aldrich (Søborg, Denmark). Phi29 reaction buffer and dNTP were from Thermo Fisher Scientific (Roskilde, Denmark).

### 2.2. DNA Oligonucleotides

DNA oligonucleotides were purchased from Microsynth Seqlab (Germany).

DNA oligonucleotides for REEAD-on-a-slide that allows combined cleavage–ligation measurement:

- 5′-amine REEAD primer: 5′-amine-CCAACCAACCAACCAAATAAG CGATCTTCACAGT- 3′;
- REEAD dumbbell substrate: 5′-AGAAAAATTTTTAAAAAAACTGTGAAGATC GCTTATTTTT TTAAAAATTT TTCTAAGTCT TTTA GATCCC TCAATGCTGC TGCTGTACTA CGATCTAAAA GACTTAGA-3′-amine;
- REEAD probe: 5′-FAM- CCTCAATGCT GCTGCT GTACTAC-3′.

DNA oligonucleotides for REEAD that allows investigation of cleavage–ligation as separate steps (REEAD (C|L)):

- 5′-amine REEAD (C|L) primer: 5′-amine-CCAACCAACCAACCAAGGAGCCAAACATGTGCATTGAGG-3′;
- Cleavage half-dumbbell: 5′-phospho-AAA AAT TTT TTC TAA GTC TTT TAC CCT CAA TGC ACA TGT TTG GCT CCG TAA AAG ACT TAG A-3′-amine;
- Ligator half-dumbbell: 5′-AGA AAA AAT TTT TAG CTC GAA CTG TGA AGA TCG CTT ATT CGA GCT-3′;
- REEAD(C|L) probe 5′-FAM ACTGTGAAGATCGCTTAT-3′.

### 2.3. Phi29 Polymerase Purification

The synthetic gene of the phi29 Polymerase was purchased from GenScript and cloned into the pGEX vector resulting in a recombinant N-term GST-tagged phi29 Polymerase expression plasmid. *E. coli* competent cells BL21 (Promega) were transformed with the plasmid and grown in 2TY media supplemented with 100 µg/mL of ampicillin. Expression of the fusion protein was induced in log phase cells at OD = 0.8, by addition of 1 mM isopropyl b-D-1-thiogalactopyranoside at 37 °C for 2 h. Cells were harvested after induction and resuspended in sonication buffer (50 mM Tris-HCL pH 7.5, 2.5 M NaCl, 1 mM EDTA, 1 mM DTT, 10 mg/mL of Lysozyme). Following 1 h of incubation on ice, the cells were then lysed by freezing and thawing in liquid N_2_ followed by sonication. After centrifugation, the lysate was mixed with 4% Streptomycin Sulfate for 1 h at 4 °C. The insoluble particles were removed by centrifugation and the lysate was filtered by using 0.45 µm filters. The lysate was loaded onto a pre-equilibrated GST Gravitrap column (#28952360, Ge Healthcare, Chicago, IL, USA) following the manufacturer’s instructions. The column was washed in 10 volumes of sonication buffer. Protein was eluted in 10 column volumes of elution buffer (Tris HCl pH 8, 5 mM Glutathione, 500 mM NaCl), and collected in fractions. The fractions were analyzed on a protein gel. The fractions were then adjusted to 50% glycerol, 0.5% Tween20, 1 mM DTT and 0.5% NP40 and stored at −20 °C.

### 2.4. REEAD-on-a-Slide

The reactions were carried out onto primer-coupled high density (HD) glass slides (#DHD1-0023 Surmodics, Eden Prairie, MN, USA). Then, 25 mm^2^ squared hydrophobic areas were drawn on the glass surface using a mini pap pen (#008877 Thermo Fischer, Denmark). The 5′-amine REEAD primer was coupled to the squares of the slides according to the Surmodics manufacturer descriptions. A total of 1 pmol of the REEAD substrate was hybridized to the primer-coupled squares of the slide for 60 min at 37 °C. In total, 200 fmol of TOP1, purified as previously reported [34])was incubated with the REEAD dumbbell substrate, coupled to the squares of the slide, in 3 μL standard TOP1 reaction buffer containing 10 mM Tris-HCl, pH 7.5, 5 mM CaCl_2_, 5 mM MgCl_2_, and 150 mM, or higher concentrations of NaCl as indicated, for 30 min at 37 °C. The circularization reactions were terminated by addition of 0.3% SDS. The slides were washed for one minute at room temperature in wash buffer 1 (0.1 M Tris-HCl, pH 7.5, 150 mM NaCl, and 0.3% SDS) followed by one minute at room temperature in wash buffer 2 (0.1 M Tris-HCl, pH 7.5, 150 mM NaCl, and 0.05% Tween-20). Finally, the slides were dehydrated in 99.9% ethanol for one minute and air-dried. The RCA was performed for 60 min at 37 °C in 1× phi29 buffer (50 mM Tris-HCl, 10 mM MgCl_2_, 10 mM (NH_4_)_2_S0_4_, 4 mM DTT pH 7.5) supplemented with 0.2 μg/μL BSA, 250 μM dNTP, and 1 Unit/μL phi29 DNA polymerase, according to commercial vendor guidelines. The RCA reaction was stopped by washing the slide for 10 min in wash buffer 1, followed by one minute in wash buffer 2 and one minute in 99.9% ethanol and the slide was then air-dried. The Rolling Circle Products (RCPs) were detected by hybridization of 0.17 μM of REEAD fluorescent probe in a buffer containing 20% formamide, 2× SSC (300 mM NaCl, 30 mM sodium citrate) and 5% glycerol for 30 min at 37 °C. The slides were washed for one minute in wash buffer 1 followed by one minute in wash buffer 2, dehydrated with 99.9% ethanol, mounted with Vectashield (#H-100 Vector laboratories, Burlingame, CA, USA), and visualized in the Olympus IX73 fluorescent microscope. Fifteen pictures for every square of the slide were taken using a 63× objective and the TOP1 activity was quantified counting the fluorescent dots using the ImageJ software.

### 2.5. T4 DNA-Ligase Purification

The synthetic gene of the T4 DNA-ligase was purchased from GenScript and cloned into the pQE-1 expression vector resulting in a recombinant N-term His6 tagged T4 DNA-ligase expression plasmid. Competent *E. coli* BL21 cells (Promega) were transformed with the plasmid and grown in 2TY media supplemented with 25 µg/mL of chloramphenicol. Expression of the fusion protein was induced in log phase cells at OD = 0.8, by addition of 0.4 mM isopropyl b-D-1-thiogalactopyranoside at 28 °C. Cells were harvested 18 h after induction and resuspended in sonication buffer (50 mM Na_2_HPO_4_/NaH_2_PO_4_, pH 8.0, 0.1% Tween20, 10% glycerol and 20 mM imidazole). The cells were then lysed by sonication and insoluble particles removed by centrifugation. The lysate was filtered by using 0.45 µm filters, 500 mM NaCl was added and the lysate and applied to a pre-equilibrated Sepharose column. Protein was eluted in 10 column volumes of sonication buffer with 300 mM NaCl and collected in fractions. The fractions were analyzed on a protein gel and dialyzed against dialysis buffer (20 mM Tris–HCl pH 7.0, 40 mM NaCl, 1 mM DTT and 20% glycerol). The fractions were then adjusted to 50% glycerol and stored at −20 °C.

### 2.6. REEAD (C|L)

The reactions were carried out onto primer-coupled HD glass slides as described for the REEAD-on-a-slide. The 5′-amine REEAD (C|L) primer was coupled to the squares of the slides according to the Surmodics manufacturer descriptions. In total, 1 pmol of cleavage-half-dumbbell substrate was hybridized to the primer-coupled slides. To measure the effect of small-molecule compounds on the binding/cleavage step of TOP1 catalytic cycle, 200 fmol of purified TOP1 [34] was added to the cleavage half-dumbbell-coupled squares of the slide in 3 μL of a standard TOP1 reaction buffer containing 10 mM Tris-HCl, pH 7.5, 5 mM CaCl_2_, 5 mM MgCl_2_, 100 mM NaCl for 30 min at 37 °C and in the presence of 5% DMSO, or 50 μM CPT or 50 μM compound POTHQ [33]. Note, the substrate was added in an approximate five-time surplus compared to enzyme. Since, the enzyme was consumed in this dead-end reaction this ensured sufficient surplus of substrate in the duration of the experiment for the potential inhibitory effect of the added compounds to be measured. The slides were then washed twice for three minutes with a buffer containing 10 mM Tris-HCL pH 7.5 and 1 mM EDTA to remove all traces of DMSO, CPT or compound POTHQ. Subsequently, 3 μL standard TOP1 reaction buffer with 200 pmol ligator-half dumbbell and 500 mM NaCl was added to the squares of the slide and incubated for 60 min at 37 °C. The slides were then washed for one minute in wash buffer 1, one minute in wash buffer 2 and one minute in 99.9% ethanol as described in the REEAD-on slide. The circularization reactions were completed by the addition of 10 Unit/μL of T4 DNA-ligase in a buffer containing 50 mM Tris-HCl pH 7.5, 10 mM MgCl_2_, 1mM ATP for 60 min at 25 °C. The slides were washed in wash buffers 1 and 2 and dehydrated. RCA was performed as described in the REEAD-on-a-slide and the RCPs were detected by hybridization to 0.17 μM of REEAD (C|L) probe in a buffer containing 20% formamide, 2× SSC (300 mM NaCl, 30 mM Sodium citrate) and 5% glycerol for 30 min at 37 °C. RCPs were visualized and quantified as described in the REEAD-on-a-slide [22].

For the measurement of the inhibition of the ligation step of the TOP1 catalytic cycle, cleavage was performed as described above, but in the absence of any added inhibitor. Subsequently, 3 μL of standard TOP1 buffer with 200 pmol ligator-half dumbbell and 500 mM NaCl was added to the squares of the slide in the presence of 5% DMSO, 50 μM CPT or 50 μM compound POTHQ [29] and incubated for 10 min at 37 °C. Note that the incubation time was estimated by the reaction time of similar ligation reactions performed previously by us and others [17,22]. The circularization and rolling circle amplification were completed as described for the measurement of the inhibition of the binding/cleavage.

### 2.7. Colorimetric REEAD-on-a-Slide Using Silver-on-Gold Precipitation

The reactions were performed onto REEAD-primer coupled HD glass slides assembled into a plastic multiwell. The multiwell was designed and printed in polyethylene terephthalate glycol (PETG) using a Prusa i3 MK3S 3-D printer. Then, 0.1 μM of REEAD dumbbell substrate was hybridized to the REEAD-primer coated slide in each well. Serial dilution of purified TOP1 from 38.5 to 2.4 μM were added and the circularization was carried out for 60 min at 37 °C. Following the circularization, the slides were washed as described for the REEAD-on-a-slide. The RCA was performed for 120 min at 37 °C in 1x phi29 buffer (50 mM Tris-HCl, 10 mM MgCl_2_, 10 mM (NH_4_)_2_SO_4_, 4 mM DTT pH 7.5) supplemented with 0.2 μg/μL BSA, 250 μM dNTP, 20 μM biotin-dCTP (# NU-809-BIO16 Jena Biosciences, Jena, Germany), and 1 Unit/μL of phi29 DNA polymerase. The RCA reaction was stopped by washing the slide for 10 min in wash buffer 1, followed by one minute in wash buffer 2 and one minute in 99.9% ethanol and the slide were then air-dried. A total of 5 μL of streptavidin-coated gold nanoparticles (Sigma-Aldrich #S9059, Søborg, Denmark) were added into the wells in PBS buffer supplemented with 2.5 mg/mL of BSA and 0.05% Tween 20 and incubated for 60 min at 25 °C. The wells were then washed twice for 5 min in PBS with 0.05% Tween20 followed by one wash in ddH_2_0 for one minute. The silver precipitation on the gold nanoparticles was achieved by using the silver staining kit (Sigma-Aldrich #SE100) following the manufacturer’s instructions. The slide was then washed in ddH20 and scanned into a Geldoc instrument (Bio-rad, Hercules, CA, USA). The squares of the slide were subjected to densitometry and quantified using the ImageJ software.

### 2.8. Statistic

Data were analyzed using GraphPad Prism software and expressed as average ± SD. When reported, Student’s *t*-test was performed and the *p*-values determined and reported in the figures.

## 3. Results

### 3.1. REEAD-on-a-Slide and REEAD (C|L) Assay Setups

**Figure 1:**
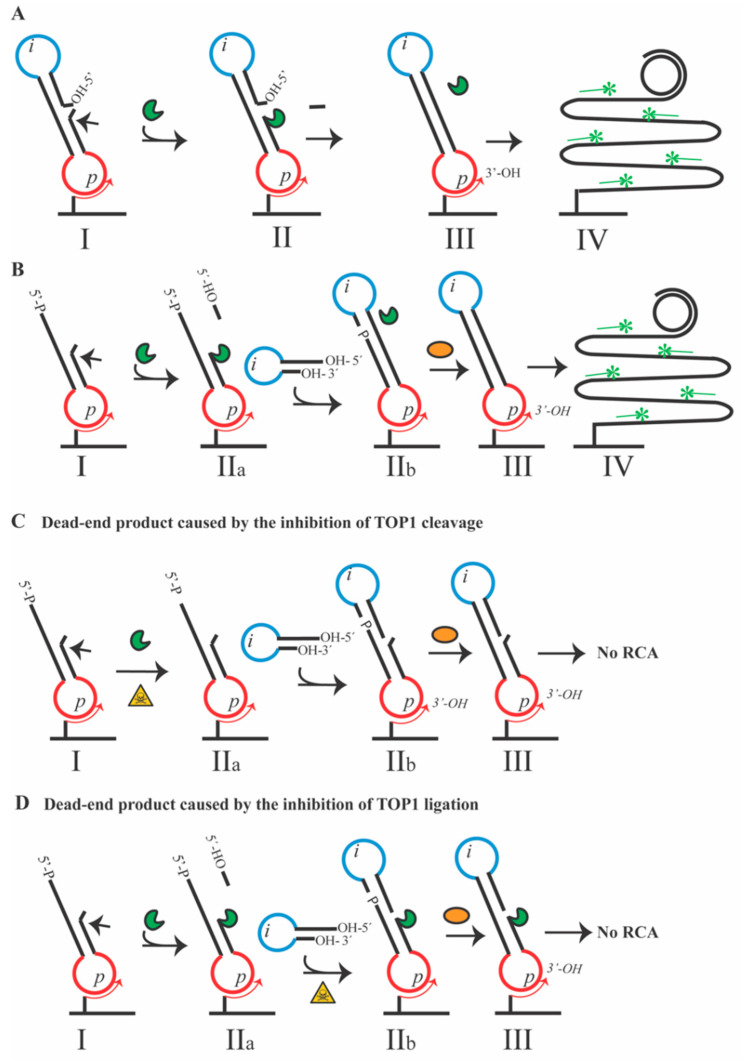
Schematic representation of the REEAD-on-a-slide and REEAD (C|L) assay setup. (**A**) The REEAD substrate folds into a dumbbell-shaped structure with one loop (red) containing a sequence matching the sequence of a primer attached to a glass slide and the other loop (blue) containing a sequence allowing hybridization of the RCP to a specific probe. The substrate contains a preferred TOP1 cleavage site (indicated by an arrow) and has a 5′-OH end suitable for TOP1 mediated ligation (I). Upon cleavage, a three-nucleotides fragment is released and diffuses away (II) while the 5′-OH is ligated by TOP1. Thereby, the open substrate is converted into a closed circle (III). The generated circle is amplified by RCA, mediated by the phi29 polymerase, and primed by the 3′-OH end of the primer (III–IV). Fluorescently labeled probes are hybridized to the RCP allowing for the visualization in a fluorescence microscope (IV). (**B**) The cleavage half-dumbbell substrate folds into a partially double-stranded molecule with one loop (red) containing a sequence allowing hybridization to a surface-attached primer. The preferred TOP1 cleavage site is situated three bases upstream of the 3′end of the half-dumbbell structure, indicated by an arrow (I). Upon cleavage, TOP1 is covalently bound to the 3′end of the cleavage half-dumbbell and ligation is prevented by the diffusion of the three-nucleotides fragment generated during cleavage and the 5′-P of the cleavage half-dumbbell (IIa). The ligation is initiated by the addition of a ligator half-dumbbell substrate (IIb) containing a 5′-OH end and a loop region (blue) allowing hybridization of probes to the subsequently generated RCP. The T4 DNA-ligase is added to ensure the ligation of the 5′-P end of the cleavage half-dumbbell and the 3′-OH of the ligator half-dumbbell (III). The two ligation events, mediated by TOP1 and T4 DNA-ligase convert the two half-dumbbell substrates into a closed DNA circle (III), which is amplified by RCA and visualized as described in A. (**C**) Measurement of inhibitor effect on the binding/cleavage step of TOP1 catalysis. The cleavage half-dumbbell substrate is hybridized to a surfaced-attached primer as described in (**B**) with the preferred TOP1 cleavage site indicated by an arrow (I). In the presence of a binding/cleavage inhibitor, the half cleavage dumbbell cannot be cleaved and TOP1 is subsequently washed away (IIa). The ligator half-dumbbell is added (IIb) and it is ligated to the cleavage half-dumbbell by the T4 DNA-ligase (III) leaving a nick that will not allow the RCA. (**D**) Measurement of inhibitor effect on the ligation step of TOP1 catalysis. The cleavage half-dumbbell substrate is hybridized to a surfaced-attached primer as described in (**B**) with the preferred TOP1 cleavage site indicated by an arrow (I). Upon cleavage, TOP1 is covalently bound to the 3′end of the cleavage half-dumbbell and ligation is prevented by the diffusion of the three-nucleotides fragment generated during cleavage and the 5′-P of the cleavage half-dumbbell (IIa). The inhibitor under investigation is added before the ligation is initiated by the addition of a ligator half-dumbbell substrate (IIb) containing a 5′-OH end and a loop region (blue) allowing hybridization of probes to the subsequently generated RCP. In the presence of a ligation inhibitor, TOP1 stays covalently bound to the cleavage half-dumbbell (IIb). The addition of the T4 DNA-ligase ensures the ligation of the 5′-P end of the cleavage half-dumbbell and the 3′-OH of the ligator half-dumbbell (III). The absence of the TOP1 ligation event prevents the conversion of the two half-dumbbell substrates into a closed DNA circle (III), and the subsequent amplification by RCA. Green pacman: TOP1, orange oval: T4 DNA-ligase; green asterisks: fluorescent probes; p: primer binding sequence; i: identifier sequence allowing hybridization of probes to the generated RCP. Yellow triangle with a skull: compound under investigation.

As mentioned in the introduction, we previously described the so-called REEAD-on-a-slide assay [24] that allows hypersensitive and quantitative measurement of TOP1 activity even in crude biological samples. The setup is depicted in Figure 1A and is composed of a single stranded DNA substrate that folds into a dumbbell-shaped structure hybridized to a surface-attached primer with a sequence matching the *p*-site of the substrate. The dumbbell substrate creates a cleavage site for TOP1 (indicated by an arrow) and places the 5′-OH end correctly to facilitate ligation (Figure 1A(I)). Following cleavage and covalent attachment of TOP1 to the substrate (Figure 1A(II)), TOP1 will immediately ligate the 5′-OH end of the substrate and generate a closed DNA circle (Figure 1A(III)). The circle is then subjected to RCA primed by the 3′-OH of the primer and catalyzed by phi29 polymerase. RCA generates an ~10^3^ tandem repeat product (Figure 1A(IV)), which can be detected at the single molecule level as a fluorescent rolling circle product (RCP) (labeled by hybridization with a fluorescent probe with a sequence matching the *i*-site) that appears as a fluorescent dot in a fluorescence microscope. The individual dots can be captured using a microscope camera and quantified. This assay presents a method to screen for the effect of small-molecule compounds with potential anti-TOP1 activity. However, the assay does not allow the effect of the compounds on the separate binding/cleavage–ligation steps of TOP1 catalysis to be dissected, since ligation follows immediately after cleavage in the REEAD. For this reason, we developed a modified version of the REEAD-on-a-slide setup, the REEAD (C|L), that allows the effect of small-molecule compounds on the separate TOP1 cleavage and ligation steps to be measured. This is accomplished by using a partly double-stranded substrate that contains only one loop of the original dumbbell substrate and has a 5′-P modification (termed cleavage half-dumbbell in the following). Like the full dumbbell substrate depicted in Figure 1A, the cleavage half-dumbbell substrate forms a TOP1 cleavage site indicated by the arrow (Figure 1B,I). However, in contrast to the full dumbbell substrate the cleavage half-dumbbell does not support TOP1 ligation in part due to the diffusion of the short three-nucleotides fragment that is cleaved off essentially as described in [17,21] and in in part due to the 5′-phosphate modification of the substrate. Consequently, TOP1 gets trapped on the substrate (Figure 1B(IIa)). The ligation can be initiated by the addition of a ligator half-dumbbell substrate with a 5′-OH end and a sequence complementary to the single stranded part of the cleavage half-dumbbell (Figure 1B(IIb)). In this sense, the step II of the original REEAD-on-a-slide assay depicted in Figure 1A, is divided in two steps (Figure 1B(IIa,IIb)). This allows the effect of small-molecule compounds on each of these steps to be investigated separately simply by adding the compounds either at the binding/cleavage step of the reaction (depicted in Figure 1C) or at the ligation step (depicted in Figure 1D). TOP1 is not able to ligate the 5′-P end of the cleavage half-dumbbell substrate. Hence ligation of this site, which is necessary to generate a closed circle that can support RCA and subsequent visualization (Figure 1B(IV)), has to be facilitated by the addition of T4 DNA-ligase (Figure 1B(III)). Note, however, that T4 DNA-ligase will not be able to ligate the 5′-OH end of the ligator half-dumbbell and the generation of signals will, therefore, strictly depend on TOP1 cleavage–ligation activity even in the presence of T4 DNA-ligase.

### 3.2. REEAD (C|L) Can Be Used to Investigate TOP1 Binding/Cleavage and Ligation Separately

**Figure 2:**
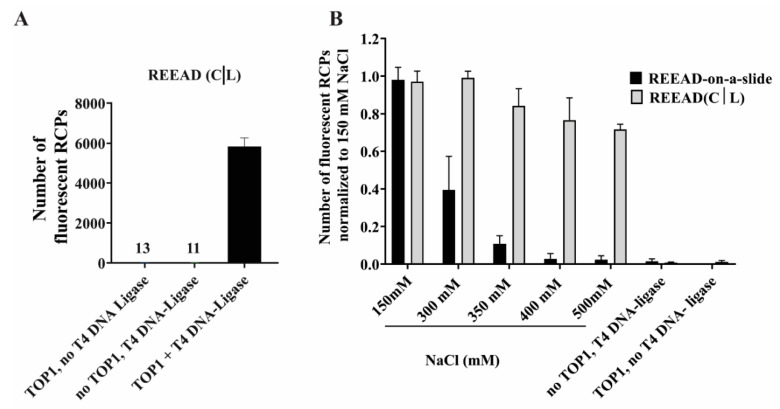
REEAD (C|L) enables separate investigation of the binding/cleavage or the ligation steps of the TOP1 catalytic cycle. (**A**) Graphical depiction of the number of fluorescent RCPs obtained by REEAD (C|L) performed in the absence of T4 DNA ligase or TOP1 or in the presence of both enzymes. The error bars represent the average of three independent experiments. (**B**) Salt titration of the REEAD-on-a-slide or the REEAD (C|L). In the REEAD-on-a-slide assay (black bars), increasing NaCl concentrations (as indicated) were added during the incubation of TOP1 with the dumbbell substrate. In the REEAD (C|L) (grey bars) increasing NaCl concentrations (as indicated) were added specifically during the ligation step. Two negative controls were included, one without TOP1 for both REEAD and REEAD (C|L) and one without T4 DNA-ligase for REEAD (C|L). The number of fluorescent RCPs was counted using Image J and normalized to the highest number of fluorescent RCPs obtained at 150 mM NaCl. Error bars represent standard deviation of four independent experiments.

As illustrated in Figure 1B, the REEAD (C|L) relies on joining of two different half-dumbbell substrates into a circle by the action of TOP1 in combination with a T4 DNA-ligase. The joining of the two half-dumbbells brings together in a single circle, (1) the primer-annealing site (*p*) necessary for priming of RCA and (2) the identifier site (*i*) necessary for visualization of the generated RCP by hybridization with a fluorescent probe. However, the assay can only be regarded as a separated TOP1 binding/cleavage–ligation activity assay, if TOP1 activity is indeed necessary for generation of signals and if these rely on the combination of the two dumbbells as depicted in Figure 1B. To test the functionality of the assay setup, the cleavage-half dumbbell was therefore incubated with or without TOP1 before the ligator half-dumbbell was added with or without subsequent addition of T4 DNA-ligase. The number of signals generated in each sample were quantified and the results depicted as a bar chart in Figure 2A. As evident, signals were only obtained when both TOP1 and T4 DNA-ligase were added and not in the presence of either one of the enzymes alone. This was also the case when signals were detected in a probe-independent manner by incorporation of α-^32^P-ATP (data not shown). Taken together the obtained results strongly support that the signals can only be generated by the fusion of the cleavage- and the ligator half-dumbbells by the combined action of TOP1 and T4 DNA-ligase as anticipated. Hence, unspecific ligation by T4 DNA-ligase cannot generate a detectable circle. Neither can unspecific circularization of the cleavage half-dumbbell by TOP1, which could at least theoretically be envisioned due to e.g., incomplete phosphorylation of the 5′-OH end.

In order to enable investigations of the effect of small-molecule compounds on the individual binding/cleavage–ligation steps of TOP1 catalysis it is necessary to enable only one of the catalytic steps assayed in REEAD (C|L) at a time. Preventing the ligation step is straightforward and can be done simply by avoiding the addition of the ligator half-dumbbell. Hence, addressing the effect of small-molecule compounds on binding/cleavage can be done by adding the compound of interest in the step IIa (Figure 1C) of the assay, in which TOP1 is mixed with the cleavage half-dumbbell. Washing away the compound before the ligator half-dumbbell is added (step IIb, Figure 1C) will ensure that any effect observed can be ascribed to an effect on cleavage. Separating the cleavage from the ligation step of TOP1 catalysis is more challenging since cleavage has to precede ligation. However, previous studies have demonstrated that the cleavage step of TOP1 catalysis is considerably more salt sensitive than ligation [18,20]. In line with this, and in agreement with previous reports [35] we did observe a strong inhibition of REEAD, which measures the combined cleavage–ligation activity of TOP1, upon addition of at least 300 mM NaCl, with a complete inhibition of the reaction upon addition of 400 mM NaCl (see bar chart, Figure 2B). In contrast REEAD (C|L) was not or only moderately inhibited by up to 500 mM NaCl added to the ligation step (IIb, Figure 1B) of the reaction. This is in agreement with previous reports, demonstrating that cleavage and not ligation is prevented by the addition of high salt concentrations and that re-cleavage of a ligated TOP1 site can be prevented by NaCl concentrations of 300 mM or higher [21,35]. For the REEAD setup the lowest salt concentration that resulted in maximum inhibition of cleavage was 400 mM of NaCl, which was used in the following experiments addressing ligation separately.

### 3.3. Measurement of TOP1 Inhibition by Small-Molecule Compounds Using REEAD (C|L)

**Figure 3:**
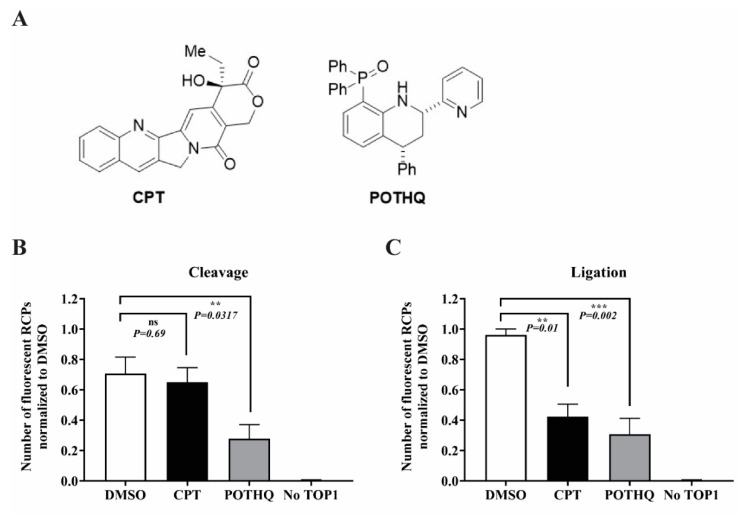
REEAD (C|L) allows the investigation of the effect of small-molecule compounds on the binding/cleavage or ligation steps of the TOP1 catalytic cycle. (**A**) Chemical structures of CPT and POTHQ. (**B**) Effect of CPT or POTHQ on the cleavage activity of TOP1 measured by REEAD (C|L). TOP1 was incubated with the cleavage half-dumbbell substrate in the presence of 5% DMSO (white bar), 50 μM CPT (black bar) or 50 μM POTHQ (grey bar), respectively. After the cleavage was completed, the compounds were washed away, and the assay completed as described. The number of fluorescent RCPs was counted using Image J and normalized to the highest number of RCPs obtained when DMSO was used. The results were plotted as average and the error bars represent standard deviation of four independent experiments. *p*-values were determined using Student’s *t*-test. ns: not significant. (**C**) Effect of CPT or POTHQ on the ligation activity of TOP1 measured by REEAD (C|L). Following cleavage, the ligation was assayed in presence of 5% DMSO (white bar), 50 μM CPT (black bar) or 50 μM POTHQ (grey bar) and the results depicted as described under B. ** *p* value ≤ 0.05. *** *p* value < 0.005.

To address if REEAD (C|L) can be used to dissect which steps of TOP1 catalysis are affected by small-molecule compounds, we measured the effect of the well-known TOP1 poison CPT [3,10] (Figure 3A, left) and the less characterized TOP1 inhibitor POTHQ [33] (Figure 3A, right) on the separate binding/cleavage and ligation reactions assayed as described above. In total, 50 μM of either of the two compounds or 5% of the solvent DMSO (corresponding to the content of the DMSO in the two test samples) were added to the reaction mixtures either during the cleavage step or during the ligation step of REEAD(C|L). Subsequently, the number of signals was quantified, and the results depicted as bar charts shown in Figure 3. As evident from Figure 3B, the addition of CPT did not affect the binding/cleavage reaction relative to the DMSO control, whereas POTHQ caused an ~50% reduction in the number of signals obtained under the assay conditions used. When the compounds were added during the ligation step, on the other hand, we did observe an ~60% reduction in TOP1 generated signals in both cases (Figure 3C). The pattern of inhibition observed for CPT is in accordance with the poison mechanism of action of this drug reported in the literature [36,37] and the ability of CPT to introduce nicks in supercoiled plasmids in the presence of TOP1 due to the selective inhibition of ligation (Supplementary Figure S1). The dual inhibition of POTHQ of both the binding/cleavage and ligation steps suggests that this compound acts as an inhibitor of the overall TOP1 activity. This is consistent with the previously reported ability of this compound to inhibit relaxation [33] and the lack of accumulation of nicks induced in supercoiled plasmid by this compound in combination with TOP1 (Supplementary Figure S1). Taken together the obtained results demonstrate the feasibility of REEAD (C|L) to dissect which steps of TOP1 catalysis are affected by a given small-molecule compound. Note, that the degree of inhibition can be analyzed in more detail by performing kinetics. However, this is not the scope of the current study that merely aims to demonstrate the applicability of the assay setup.

### 3.4. Signal Readout Using Silver-on-Gold Precipitation

**Figure 4:**
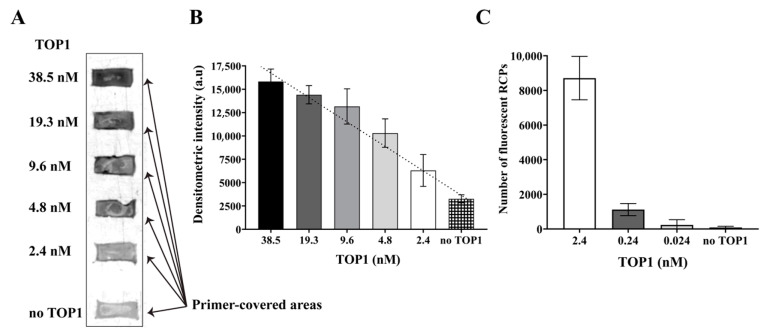
Silver-on-gold precipitation readout can be used for measuring TOP1 activity. (**A**) Scanned image obtained by the Bio-rad Geldoc of the REEAD-on-a-slide assay performed with serial dilutions of TOP1 and visualized using a silver-on-gold precipitation readout. The concentrations of TOP1 in each sample are indicated to the left. (**B**) Graphical depiction of a densitometric quantification of the scanned image shown in A. The data were plotted as average and error bars represent standard deviation of three independent experiments. a.u: arbitrary units. (**C**) Graphical depiction of the number of fluorescent RCPs obtained by measuring the activity of serial dilution of TOP1 by using the REEAD-on-a-slide. The data were plotted as average, and the error bars represent standard deviation of three independent experiments.

The REEAD (C|L) presents a convenient method for dissecting the effect of potential novel anti-parasitic or anti-cancer small-molecule drugs that target TOP1. However, the microscopic readout utilized to obtain single-molecule resolution is unnecessary for drug screening purposes. Indeed, it may be tedious and require access to a fluorescence microscope, which is not possible for all laboratory setups. Therefore, we here investigated an alternative readout based on a simple colorimetric method that does not require special equipment. In this setup, biotinylated nucleotides are incorporated during RCA. This allows for subsequent coupling of streptavidin functionalized gold nanoparticles that support silver precipitation as described in Section 2.7. The silver-on-gold precipitation results in a dark pigmentation that is directly visible by the naked eye and that can easily be documented by a cell phone camera or standard gel documentation equipment. Like the fluorescence microscope dependent readout, the silver-on-gold precipitation provides a directly quantitative measure as demonstrated by the TOP1 titration experiment shown in Figure 4A,B. In this experiment, decreasing concentrations of TOP1 ranging from 38.5 to 2.4 nM were assayed by REEAD-on-a-slide. The results were visualized using the silver-on-gold precipitation readout and quantified by densitometry (Figure 4A,B). For comparison, circles obtained by incubation of the dumbbell substrate with 2.4 to 0.024 nM of TOP1 were amplified without biotinylated nucleotides and the RCPs visualized by fluorescence microscopy and counted following standard protocol (Figure 4C). As evident from the graphical depiction of the results (Figure 4B,C), both readout methods showed a linear relationship with the TOP1 concentration. The detection limit of the fluorescent readout was 20 orders of magnitudes better than that of the colorimetric readout (compare Figure 4B,C). However, for drug screening purposes using cell extracts or (partly) purified TOP1 this should not matter and can be adjusted for, simply by adding more enzyme.

## 4. Discussion and Conclusions

We here present an easy-to-use assay that allows for screening of TOP1 targeting small-molecule compounds and dissect which steps of TOP1 cleavage–ligation activity are affected. The identification of novel TOP1 targeting compounds with potential clinical activity is of interest both in the combat of infectious diseases and for treatment of cancer. Indeed, the TOP1 specific drugs irinotecan and topotecan are among the most promising drugs when it comes to treatment of colon-, ovarian and small cell lung cancers that fail to respond to other anti-cancer therapy [8,9,10]. However, even after decades of use, these drugs remain the only FDA approved TOP1 targeting anti-cancer drugs. When surgery is not an option, cancer remains a highly deathly disease and the identification of new drugs, including TOP1 targeting drugs is of immense need.

Additionally, for treatment of infectious diseases the identification of new effective and specific drugs is in high demand. This is not least the case for eukaryotic pathogens in which the potential drug targets often have human homologs making the identification of drugs with few or no side effects particularly challenging [2,38].

As mentioned, TOP1 activity appears to be essential for most single cell pathogens but not for mammals except in the early developmental stages. Hence, TOP1 targeting anti-cancer drugs are only effective in killing the human cancerous cells if they turn TOP1 activity into a cell poison by inhibiting the ligation step and causing accumulation of TOP1 coupled DNA damage in the cell [9,10,38]. Decreasing the overall TOP1 activity has little or no effect on the fitness of human cells. This, on the contrary, can kill human pathogens making drugs that inhibit the DNA binding and cleavage steps of TOP1 attractive possibilities for future anti-parasitic treatment with minimal side effects for the human host.

For the above reasons, the identification of novel small-molecule compounds that will affect either (1) the DNA binding and cleavage or (2) the ligation of TOP1 catalysis is in high demand. Unfortunately, screening for new compounds with such activities is rather troublesome and requires both equipment and skills that are not available in all laboratories, especially not in laboratories focusing on the synthesis of small-molecule compounds. This delays the development of new drugs to a large extent. To overcome such obstacles, we here present and demonstrate the functionality of a novel assay, the REEAD (C|L), for dissecting the inhibitory activity of small-molecule compounds. This was done by using the well-known anti-cancer compound CPT, and the novel TOP1 inhibitor POTHQ as examples. In agreement with previous reports using numerous different assays [16,39], we showed that CPT inhibited specifically the ligation step and not the binding/cleavage step of the REEAD (C|L), while POTHQ inhibited both the binding/cleavage and the ligation reaction. This is in agreement with previous reports of POTHQ inhibiting TOP1 relaxation [33] and the inability of this drug to induce TOP1 dependent nicking of a DNA plasmid observed in the present study. Following the original protocol of the REEAD procedure the results were obtained using the single-molecule resolution readout presented by counting of fluorescent signals in a microscope. This gives the immediate advantage of high sensitivity and very precise quantification suitable for the initial fast assessment of new compounds. However, this procedure requires special skills and equipment and presents some of the disadvantages that are also associated with traditional gel-based assays. We therefore present an easy colorimetric readout based on silver-on-gold precipitation that may be used as an alternative to the state-of-the-art microscopic readout for many purposes.

In the current study, we demonstrated the applicability of the REEAD (C|L) for measuring drug effects on purified TOP1. However, we previously demonstrated the functionality of the REEAD assay for measuring TOP1 activities in crude biological samples simply by chelating divalent cations, necessary for unspecific nuclease activities by the addition of EDTA. Even though Mg^2+^ and Ca^2+^ stimulate TOP1 mediated cleavage [21] divalent cations are not a prerequisite for TOP1 activity [40] and, hence, REEAD (C|L) will allow specific measurement of the binding/cleavage and ligation reactions of TOP1 in crude samples using an EDTA containing buffer as previously described for REEAD [22].

Taken together, our results demonstrated that REEAD (C|L) allows the effect of small-molecule compounds on the DNA binding/cleavage or the ligation step of TOP1 catalysis to be investigated separately. We believe the setup will be usable for a large number of researchers focusing on the development of novel small-molecule drugs, regardless their background and training and thereby speed up the identification of new TOP1 drugs with anti-cancer or anti-parasitic effects.

## 5. Patents

The authors C.T., M.S. and B.R.K. declare that they are named inventors on the pending unpublished patent application EP21164120 relating to the REEAD(C|L) technology described in here, filed in the name of VPCIR Biosciences ApS.

## Supplementary Materials

The following are available online at https://www.mdpi.com/article/10.3390/pharmaceutics13081255/s1, Figure S1: Nicking experiment. TOP1 was incubated in the absence (DMSO) or presence of either 50 μM CPT or 50 μM POTHQ. TOP1 was incubated with supercoiled DNA plasmid and the reactions terminated at the indicated time points. The DNA molecules were separated by gel electrophoresis on a 1% agarose gel in the presence of 0.5 μg/mL of ethidium bromide. C, negative control (supercoiled DNA only).

## Abbreviations

Topoisomerase 1 (TOP1); Topotecan (TPT); Electrophoretic Mobility Shift Assay (EMSA); Rolling circle Enhanced Enzyme Activity Detection (REEAD); Camptothecin (CPT); (4-Phenyl-2-(4-trifluoromethylphenyl)-1,2,3,4-tetrahydroquinolin-8-yl) diphenylphosphine oxide (POTHQ); Rolling Circle Amplification (RCA); Rolling Circle amplification Products (RCPs).
