# Supplementary material for: Simple and Fast DNA Based Sensor System for Screening of Small-Molecule Compounds Targeting Eukaryotic Topoisomerase 1
Source: Pharmaceutics. 2021 Aug 13;13(8):1255. doi: 10.3390/pharmaceutics13081255 (PMC8401307; doi:10.3390/pharmaceutics13081255)
Supplement: Supplementary file 1 [file pharmaceutics-13-01255-s001.zip › pharmaceutics-1300631-supplementary.pdf]

pharmaceutics

Supplementary Materials: Simple and Fast DNA Based Sensor System for Screening of Small-Molecule Compounds Targeting Eukaryotic Topoisomerase 1

Kamilla Vandsø Petersen, Asier Selas, Kirstine Mejlstrup Hymøller, Karol Mizielinski, Maria Thorsager, Magnus Stougaard, Concepcion Alonso, Francisco Palacios, Yolanda Pérez-Pertejo, Rosa M. Reguera, Rafael Balaña-Fouce

Supplementary S1

Effect of CPT or POTHQ on the TOP1 activity measured by the state-of-the-art nicking assay

The effect of the small-molecule compounds CPT and the POTHQ was investigated using the state-of-the-art agarose gel-based assay called nicking assay. TOP1 can remove supercoils from plasmid DNA by making a transient nick, which is rapidly resealed by the enzyme. In the nicking assay, TOP1 is incubated with a negatively supercoiled DNA plasmid in the absence or presence of the small-molecule compound to be investigated. After incubation, the reaction is terminated by the addition of SDS and proteinase K and the DNA plasmid is run into an agarose gel in the presence of the DNA intercalator, ethidium bromide. Due to the ethidium bromide-induced DNA unwinding effects, positive supercoils will be introduced into relaxed intact plasmid as well as in negatively supercoiled intact plasmid. This will cause the negatively supercoiled plasmid to run slightly slower in the gel compared to relaxed plasmid. The mobility of nicked plasmid, however, will not be affected by ethidium bromide since one strand is free to rotate. Thereby, the positive supercoils introduced by ethidium bromide are quickly removed. This causes nicked plasmid to run slowly in the gel and allows easy visualization of an increase in this topoisomer as a result of a TOP1 poison causing accumulation of cleavage complexes by stimulating cleavage, inhibiting ligation or both. A typical example of a small-molecule compound causing accumulation of TOP1 dependent nicks in plasmid DNA is CPT that acts by inhibiting the ligation reaction of catalysis [1,2].

As shown in Supplementary Figure S1, CPT increased the amount of nicked DNA (compare lanes 4-5 with lanes 2-3). This is consistent with CPT inhibiting the religation step of the TOP1 catalytic cycle without inhibiting the cleavage activity, thus causing an increase of the amount of nicked DNA molecules, with TOP1 covalently bound to them. On the other hand, POTHQ did not increase the amount of the nicked DNA (compare lanes 6-7 with lanes 2-3). This is consistent with POTHQ inhibiting all steps of TOP1 catalysis and thus preventing the generation of TOP1-DNA cleavage complexes and the lack of nicked DNA molecules.

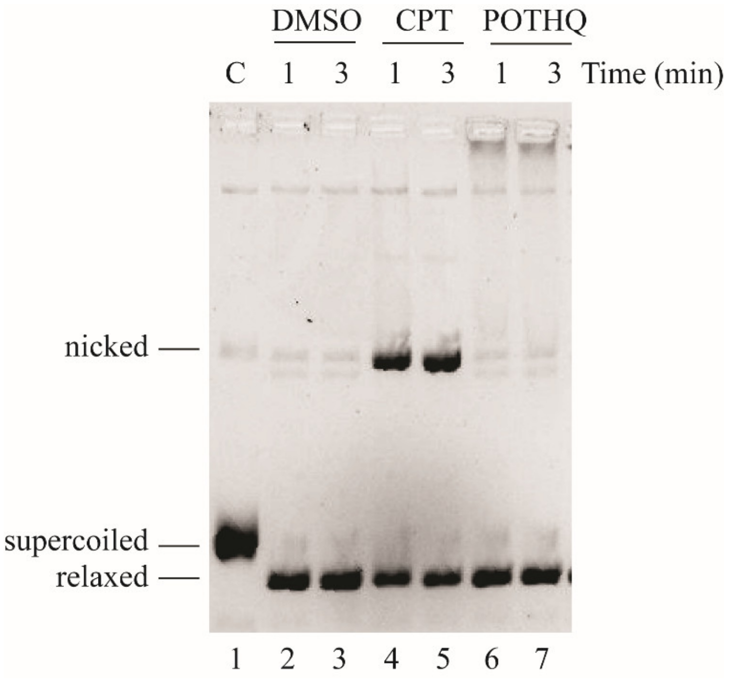

Figure S1. Nicking experiment. TOP1 was incubated in the absence (DMSO) or presence of either 50 µM CPT or 50 µM POTHQ. TOP1 was incubated with supercoiled DNA plasmid and the reactions terminated at the indicated time points. The DNA molecules were separated by gel electrophoresis on a 1% agarose gel in the presence of 0.5 µg/mL of ethidium bromide. C, negative control (supercoiled DNA only).

Supplementary materials and methods

Nicking assay

TOP1 activity was measured using a DNA nicking assay by incubating 330 ng of TOP1 with 0.5 µg of negatively supercoiled pUC18 in 20 µL of reaction buffer (20 mM Tris-HCl, 0.1 mM EDTA, 10 mM MgCl2, 10 mM CaCl2 and 150 mM NaCl, pH 7.5) and in presence of 0.1 % DMSO or 50 µM CPT or 50 µM POTHQ. The reactions were performed at 37 °C and stopped by the addition of 0.5% SDS after one or three minutes. The samples were protease digested, electrophoresed in a horizontal 1% agarose gel containing 0.5 µg/mL ethidium bromide in TBE buffer (50 mM Tris, 45 mM boric acid, 1 mM EDTA) at 26V for 20 h. The picture was acquired using a Geldoc imager (Bio-Rad).

References

[1] B.L. Staker, K. Hjerrild, M.D. Feese, C.A. Behnke, A.B. Burgin, L. Stewart, The mechanism of topoisomerase I poisoning by a camptothecin analog, Proc. Natl. Acad. Sci. U. S. A. 99 (2002) 15387–15392. doi:10.1073/pnas.242259599.
[2] C. Bailly, DNA relaxation and cleavage assays to study topoisomerase I inhibitors, Methods Enzymol. 340 (2001) 610–623. doi:10.1016/S0076-6879(01)40445-9.
